# Supplementary material for: IL-1 Receptor Contributes to the Maintenance of the Intestinal Barrier via IL-22 during Obesity and Metabolic Syndrome in Experimental Model
Source: Microorganisms. 2024 Aug 20;12(8):1717. doi: 10.3390/microorganisms12081717 (PMC11357463; doi:10.3390/microorganisms12081717)
Supplement: Supplementary file 1 [file microorganisms-12-01717-s001.zip › microorganisms-3124923-supplementary.pdf]

SUPPLEMENTARY MATERIAL

Table S1. Diet Composition (Standard diet - AIN93 M)

Product	Supplier	Batch	Prescribed quantity	Product quantity (g/ml)
Maize starch	Ingridion	180132	46,57%	3.725,54
Casein	SB	51733	14,0%	1.120,00
Dextrinized starch	Ingridion	1746919	15,5 %	1.240,00
Sucrose	GA	2109618	10,0%	800
Soy oil	LDF	420	4,0%	320
Microcrystalline cellulose	Inlab	858073	5,0%	400
Mineral mix AIN 93 M	PragSoluções	50122	3,50%	280
Vitamin mix AIN 93 M	PragSoluções	220222	1,0%	80
L cistin	Inlab	854895	0,18%	14,4
Choline bitartrate	Valdequimica	36354	0,25%	20
BHT	Oficinalls	Chasmy0610	0,0008%	0,064

Table 2. Diet composition (High Fat Diet)

Product	Supplier	Batch	Prescribed quantity	Product quantity (g/ml)
Casein	SB	51733	25,9%	3.412,90
L cistin	Inlab	854895	0,39%	51,48
Dextrinized starch	Ingridon	1746919	16,15	2.131,80
Sucrose	GA	2109618	8,9%	1.174,80
Soy oil	LDF	420	3,2%	426,36
Lard	Estrela	120222	31,7%	4.179
Macrocrystalline cellulose	Inlab	858073	6,5%	852,72
Mineral mix PSB10026	PragSoluções	50122	1,29%	170,26
Dibase calcium phosphate	Inlab	853261	1,7%	221,75
Calcium carbonate	SM	280421	0,71%	93,72
Potassium citrate	Organic	SR20B00311	2,13%	281,16
Vitamin Mix AIN 93	PragSoluções	200222	1,29%	170,28
Choline bitartate	Valdequimica	36354	0,26%	34,32
BHT	Oficinalls	Chasmy0610	0,0050%	0,66

Table 3. Primers for specific bacterial phyla or species.

Target	Forward sequence	Reverse sequence
16S	AACAGGATTAGATACCCTGGTAG	GGT TCT TCG CGT TGC ACT-3
Eubacteria	ACTCCTACGGGAGGCAGCAGT	ATTACCGCGGCTGCTGGC
Bacteroidota	GTTTAATTCGATGATACGCGAG	TTAASCCGACACCTCACGG
Pseudomonadota	CATGACGTTACCCGCAGAAGAAG	CTCTACGAGACTCAAGCTTGC
Bacillota	ATGTGGTTTAATTCGAAGCA	AGCTGACGACAACCATGCAC
Actinomycetota	TGTAGCGGTGGAATGCGC	AATTAAGCCACATGCTCCGCT
Verrucomicrobiota	TCAGTCAGTATGGCCCTTAT	CAGTTTTAGGATTTCCTCCGCC
Akkermansia muciniphila	CAGCACGTGAACGTGGGGAC	CCTTGCGGTTGCCTTCAGAT
Escherichia coli	CATGCCGCGTGTATGAAGAA	CGCGTAACCTCAATGAGCAAA
Ruminococcus torques	CGAAGCACTTTGCTTAGA	ACATCAGACTTGCCCATC
Clostridium leptum	GCACAAGCAGTGGAGT	CTTCCTCCGTTTTGTCAA
